# Neural Mechanisms of Inhibitory Response in a Battlefield Scenario: A Simultaneous fMRI-EEG Study

**DOI:** 10.3389/fnhum.2016.00185

**Published:** 2016-05-02

**Authors:** Li-Wei Ko, Yi-Cheng Shih, Rupesh Kumar Chikara, Ya-Ting Chuang, Erik C. Chang

**Affiliations:** ^1^Institute of Bioinformatics and Systems Biology, National Chiao Tung UniversityHsinchu, Taiwan; ^2^Brain Research Center, National Chiao-Tung UniversityHsinchu, Taiwan; ^3^Department of Biological Science and Technology, National Chiao Tung UniversityHsinchu, Taiwan; ^4^Institute of Cognitive Neuroscience, National Central UniversityTaoyuan, Taiwan

**Keywords:** electroencephalography (EEG), function magnetic resonance imaging (fMRI), inhibitory control, theta-alpha band, right temporoparietal junction (rTPJ)

## Abstract

The stop-signal paradigm has been widely adopted as a way to parametrically quantify the response inhibition process. To evaluate inhibitory function in realistic environmental settings, the current study compared stop-signal responses in two different scenarios: one uses simple visual symbols as go and stop signals, and the other translates the typical design into a battlefield scenario (BFS) where a sniper-scope view was the background, a terrorist image was the go signal, a hostage image was the stop signal, and the task instructions were to shoot at terrorists only when hostages were not present but to refrain from shooting if hostages appeared. The BFS created a threatening environment and allowed the evaluation of how participants’ inhibitory control manifest in this realistic stop-signal task. In order to investigate the participants’ brain activities with both high spatial and temporal resolution, simultaneous functional magnetic resonance imaging (fMRI) and electroencephalography (EEG) recordings were acquired. The results demonstrated that both scenarios induced increased activity in the right inferior frontal gyrus (rIFG) and presupplementary motor area (preSMA), which have been linked to response inhibition. Notably, in right temporoparietal junction (rTPJ) we found both higher blood-oxygen-level dependent (BOLD) activation and synchronization of theta-alpha activities (4–12 Hz) in the BFS than in the traditional scenario after the stop signal. The higher activation of rTPJ in the BFS may be related to morality judgments or attentional reorienting. These results provided new insights into the complex brain networks involved in inhibitory control within naturalistic environments.

## Introduction

Inhibitory control is a crucial aspect of cognitive control processes. It allows one to stop ongoing action when it is deemed inappropriate (Aron, 2007). Bari and Robbins (2013) suggested to divide inhibitory control into two categories: cognitive inhibition and behavioral inhibition. Cognitive inhibition can be defined as “the stopping or overriding of a mental process, in whole or in part, with or without intention” (MaCleod, 2007), and is usually measured by the interference task (Kipp, 2005; Leroux et al., 2006). In contrast, behavioral inhibition, which is the focus of the current study, refers to the suppression of actualizing behavioral outcome, and can be measured by the stop signal task (SST) or go/no-go task (GNGT). Both SST and GNGT use frequent go trials which require participants to perform an action (e.g., press a key button) and infrequent stop (no-go) trials which requires participants to inhibit preparative action (e.g., not to press a key button) upon receiving an additional SST or a different target stimulus (GNGT).

Previous studies have adopted either GNGT or SST to explore the neuroanatomical loci and temporal characteristics of associated brain activities with functional magnetic resonance imaging (fMRI) and electroencephalography (EEG), respectively. In the neuroanatomical domain, many studies found that the prefrontal gyrus (PFG) is important for executive control (for a comprehensive review, see Miller and Cohen, 2001). Consistent activation for response conflict, novelty, working memory (number of elements and delay) and perceptual difficulty has been observed in the inferior frontal gyrus (IFG), dorsal anterior cingulate gyrus (ACG), dorsolateral prefrontal gyrus (DLPFG), but not other frontal regions, regardless of the specific contrast task (Duncan and Owen, 2000). Aron et al. (2004) concluded that the right IFG (rIFG) was more closely related to inhibitory control because damage of the rIFG crucially affected performance in executive cognitive control paradigm, apparently by disrupting inhibition.

However, a number of studies have also proposed that the rIFG is recruited across different task conditions that require sustained attention (Shallice et al., 2008a,b; Simmonds et al., 2008). Hampshire et al. (2010) also suggests that the rIFG serves a general role in attentional control, which rapidly adapts in order to respond to relevant and salient stimuli related to inhibitory control in GNGT and SST. Hence, the suppression of an already initiated response likely depends on rIFG, yet exactly how the inhibitory function is manifested in the motor system remained to be investigated. On the other hand, Aron and Poldrack (2006) had shown that the subthalamic nucleus (STN), which is a part of the basal ganglia, may play a role to suppress the “direct” fronto-striatal pathway that is activated by response initiation and also involved the pre-supplemetary motor area (preSMA). The findings by Mostofsky et al. (2003) suggest that the preSMA appears necessary for inhibiting unwanted movements (stop or no-go condition). Based on previous studies (Aron and Poldrack, 2006; Nachev et al., 2008; Verbruggen and Logan, 2008), Duann et al. (2009) had applied Granger causality analysis in an fMRI study on stop-signal task to explore the functional connectivity of IFG and preSMA. Their study found that preSMA and primary motor gyrus (PMG) have functional interconnectivity via the basal ganalia circuitry to mediate response inhibition, whereas IFG connects with preSMA to modulate the basal ganglia circuitry. According to Duann et al. (2009), the PMG is mediated by IFG and preSMA via basal ganalia circuitry and the functional connectivity between IFG and preSMA is “bi-directional” in SST. Recently, IFG has been hypothesized to serve various functions including resolution of stimulus conflict, attentional orienting or the monitoring of behavior. Consequently, results from some studies have suggested that preSMA is more directly related to response inhibition than IFG, given its involvement in motor control (Bari and Robbins, 2013; Obeso et al., 2013; Aron et al., 2014).

Although the imaging studies are informative about the neuroanatomical loci of response inhibition in the brain, equallythe brain, equally important is how the inhibitory process evolved across time upon its inception. Huster et al. (2013) reviewed EEG studies on the response inhibition under GNGT and SST. Most empirical reports mainly examined event-related potentials (ERP), and it is commonly observed that both stop and no-go conditions evoked two different ERP components which are usually absent in the go condition: a fronto-central negativity occurring around 200–300 ms after stimulus onset (stop or no-go stimulus), followed by a positive potential with a delay of approximately 150 ms exhibiting a fronto-central to centro-parietal topography. These two components have often been conjointly referred to as the N2/P3 complex. Nevertheless, N200s and P300s were also evoked in a broad range of paradigms, including but not limited to response inhibition (e.g., SST, GNGT, Stroop task, Flanker task and Simon task; Kopp et al., 1996; Liotti et al., 2000; Falkenstein et al., 2002; Nieuwenhuis et al., 2004; Ramautar et al., 2006; Johnstone et al., 2007; Bruchmann et al., 2010).

To more specifically determine the temporal marker(s) for response inhibition, an alternative way of analyzing EEG data is through time-frequency analysis for uncovering the oscillatory components involved in inhibitory response (e.g., Herrmann et al., 2005). Basar et al. (1999) demonstrated that EEG can be investigated in the frequency domain and oscillations of specific frequencies are related to specific cognitive functions, such as alpha band (8–12 Hz) fluctuations during both sustained and directed attention (Mathewson et al., 2014). While ERP analysis generally compares latencies or magnitudes of components elicited by different conditions (e.g., go condition vs. stop or no-go conditions), in time-frequency analysis the oscillations of frequency bands associated with different conditions are usually compared. Recently, a number of studies have applied time-frequency analysis in response inhibition tasks. The most common findings from these time-frequency analyses are a burst in frontal-midline theta power for no-go and stop signal conditions as compared to the go condition between 200 and 600 ms after the no-go or stop signal presentation, which falling well into the time range of N2/P3 complex (Huster et al., 2013). In addition, Schmiedt-Fehr and Basar-Eroglu (2011) also reported activity in the delta power for the same time window using a GNGT. These time-frequency components seem to more specifically associated with response inhibition.

Most studies explored the “inhibitory network” by using stimuli with simple configuration in SST or GNGT (e.g., circle as the go stimulus, and an “X” as the stop (no-go) signal; Chang et al., 2014; Lavallee et al., 2014) to investigate the properties of the inhibitory network. How this inhibitory network for typical SST generalizes to response inhibition in more realistic scenarios remains to be investigated. The generalizability issue is not new in cognitive experiments, and not many studies have explored how well cognitive phenomena established in simple scenes can be generalized to more complex and realistic ones. Lapenta et al. (2014) used transcranial direct current stimulation (tDCS) to explore inhibitory control of EEG under food craving using realistic food picture as go signal in GNGT. They first induced the participant’s food craving by a brief movie showing scenes of food and then required the participant to complete a visual analog scale for appearance, smell and taste of the exposed food. All participants were then required to perform GNGT twice: one was performed with active tDCS at F4 and F3 (10–20 EEG coordinate system) and the other with sham tDCS. Their results indicated that tDCS reduced magnitude of frontal N2 component but enhanced the P3a component, as compared with the sham condition. Regenbogen et al. (2010) used real and virtual computer game scenarios to compare the pattern of brain activation between gamers and non-gamers. They analyzed fMRI data by contrasting different combination of conditions, including Violent vs. Nonviolent scenarios under real and virtual modality for gamers and non-gamers, respectively. The activity pattern of non-gamers under the contrast Violent vs. Nonviolent is more complex than gamers in both real and virtual scenes. More importantly, when the neural activities of real modality were compared with virtual modality between gamers and non-gamers, they found non-gamers have more activated brain regions when contrasting Violent vs. Nonviolent conditions, and when contrasting Real vs. Virtual scenes. Based on the findings above, it seems that real and virtual scenes may recruit the brain in distinct ways.

Given the paucity in literature exploring response inhibition by combining methods with high spatial and temporal resolutions, by applying time-frequency analyses, and by contrasting performance under simple vs. naturalistic scenarios, the current study aims to compare behavioral performance and neural mechanisms of inhibitory response under simple and realistic scenarios with simultaneous recording of EEG and fMRI. Scenes from a well-known shooting game “Count Strike” were adopted as the visual background in the battlefield scenario (BFS), where image of a “terrorist” holding a gun served as the go sign, and a “hostage” image as the stop signal. Besides higher extent of visual complexity, this scenario is supposed to induce stressful feelings in the participants. As a control condition, the conventional SST in which simple symbols represent go and stop signals, namely a symbol scenario (SBS), was also adopted. In order to investigate both the rapid brain dynamics and precise spatial loci of the inhibitory process, simultaneous fMRI and EEG recordings were carried out to acquire signals of brain activation from sources with high spatial and temporal resolutions, respectively. Comparing to independent recording of fMRI and EEG, simultaneous fMRI-EEG can confirm that the characterization of functional activations and frequency oscillations of brain networks are under the same experimental condition, and thus more likely the same neural networks (Mulert, 2013). The current study examined significant differences in fMRI and EEG responses associated with successful-stop (SS) vs. successful-go (SG) trials to identify inhibition-related brain activations/dynamics, and SS vs. fail-stop (FS) trials to identify error-related brain activations/dynamics (Li et al., 2006; Boehler et al., 2010; Swick et al., 2011). Based on the literature of inhibitory control reviewed above, we predict that preSMA will show fMRI activation and modulations in theta-alpha band power under both scenarios of SST. However, for the comparison between SBS and BFS, it remains an empirical question whether additional neural networks related to cognitive processing of emotional or social information, such as amygdala or middle temporal gyrus would be involved.

## Materials and Methods

### Participants

All participants (*n* = 35; mean age = 23.39; *SD* = 1.86) were right-handed, had normal or corrected-to-normal vision, and none reported history of neurological or psychiatric disorders. Each participant provided written informed consent approved by the Research Ethics Committee of the National Taiwan University prior to participation. Data from three participants were excluded from analyses due to low performance in SST (SG ratio is lower than 2SD below the group mean). Among the remaining participants, simultaneous fMRI-EEG data were successfully acquired from 11 participants, and 21 participants only have fMRI data. Therefore, the fMRI results were based on 32 datasets, whereas the EEG results were based on 11 datasets. Although there were only 11 participants for the EEG analysis, given that each participants made responses to 105 trials, the total amount of epochs is 1155. These epochs are distributed into the four conditions (SG = 705, SS = 170, FG = 92, FS = 188). We consider this amount of epochs are sufficient for our EEG analyses.

### Experimental Design

**Figure 1:**
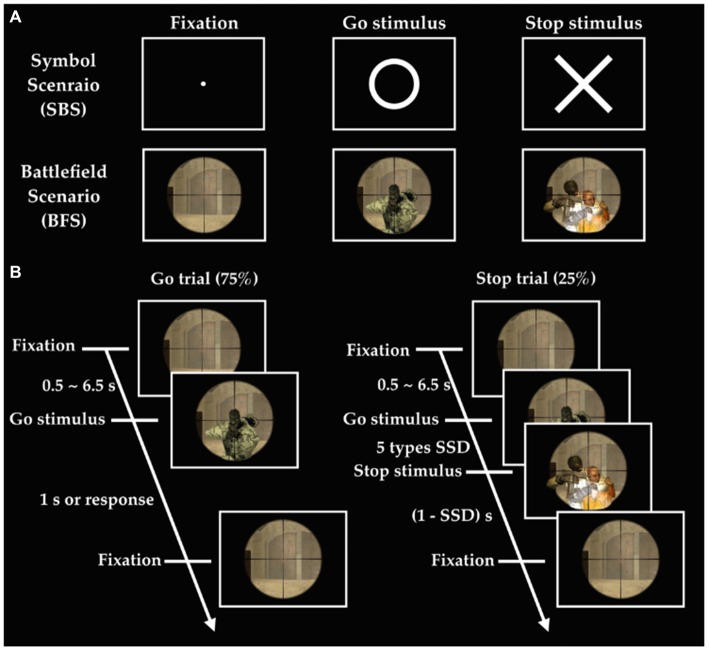
**Experimental design. (A)** Stimuli in two scenarios; **(B)** order of events in the battlefield scenario (BFS). SSD indicates stop signal delay, and there are five different SSDs including SSD (cSSD), cSSD ± 40 ms, and cSSD ± 80 ms.

The experiment implemented the stop-signal task under two different scenarios (Figure 1), where one consisted of simple symbol (i.e., SBS) and the other battlefield images (i.e., BFS). Every participant was asked to respond to a go stimulus (a circle for SBS and a ruffian for BFS). They hold their response (stop stimulus), when appeared (a cross for SBS and a hostage for BFS), when it was presented after the go stimulus. The critical stop signal delay (cSSD), which is approximately 50% probability of SS, was measured by using a staircase tracking procedure before they performed formal experimental trials in the scanner. The staircase tracking procedure worked in the following way: SSD started at 150 ms and if the participant successful-stopped their response, SSD would increase by 50 ms; on the contrary, SSD would reduce 50 ms and the lower bound of SSD was 150 ms. The formal task used five different SSDs (cSSD, cSSD ± 40 ms, and cSSD ± 80 ms) and each SSD had equal number of trials. The participant performed four runs in the fMRI experiment and each run was equally divided into one half for BFS and the other for SBS, where the order of scenario was completely counterbalanced across runs. Each block of scenario in a run had 105 trials of which 25% were stop trials while the rest were go trials. Each go trial began with a fixation cross lasting for a random duration (0.5–6.5 s), followed by a go-signal lasting for 1 s or until response. In a stop trial, the stop-signal is presented *N* milliseconds after the go-signal, where *N* was defined by the SSD assigned to that trial.

### fMRI Signal Acquisition and Preprocessing

Participants performed the task in a Siemens 3T MAGNETOM Skyra scanner located in the Taiwan Mind and Brain Imaging Center at National Chengchi University, Taipei. Structural T1-weighted images were acquired using the MPRAGE sequence (TR: 2530 ms; TE: 3.03 ms; flip angle: 7°; matrix size: 224 × 256; field of view: 224 × 256 mm; in-plane resolution: 1 × 1 mm; slice thickness: 1 mm; 192 slices). Functional brain images were acquired using a gradient echo-planar imagine sequence (TR: 2000 ms; TE: 25 ms; flip angle: 90°; matrix size: 64 × 64; field of view: 220 × 220 mm; voxel size: 3.438 × 3.438 × 4.0 mm^3^; 292 volumes per run). The preprocessing stream as well as statistical analyses was completed using the Analysis of Functional Neuroimages (AFNI) software (Cox, 1996). The preprocessing stream included image reconstruction, slice-time correction (time-shifting the time series using Fourier interpolation), and motion-correction (linear least-squared alignment via affine transformation with three translational and three rotational parameters). Activation outside the brain was removed using edge detection techniques. After the preprocessing, each participant’s anatomical image was transformed into the standard space of the Montreal Neurological Institute (MNI) 152 brain template using an automated feature-matching algorithm (Collins et al., 1994). Each participant’s functional data was first aligned to their own anatomical image and then transformed into the standardized MNI space.

### EEG Signal Acquisition and Preprocessing

An MR-compatible 34-channel amplifier (BrainAmp MR; Brain Products) and a MR-compatible EEG cap (BrainCap-MRI 32-Channel-Standard) with a head volume coil were applied in this study. EEG was recorded in the MR scanner room simultaneously with fMRI acquisition. The EEG cap had 31 electrodes for brainwave recording and one for electrocardiography (ECG) recording. Electrode-skin impedance was kept smaller than 10 kOhms by using abrasive electrolyte-gel (ABRALYT HiCl). Data were transferred through fiber-optic cables to an IBM-compatible laptop and recorded by the BrainVision Program (BrainVision Recorder, Brain Products) synchronized with the BOLD signals via triggers from the MR scanner. The EEG signals were recorded with a passband of 1–250 Hz, digitized at 5000 Hz with 32-bit of resolution (equivalent to 0.5 μV; dynamic range: 16.38 mV). The EEG data were band-pass (1–50 Hz) filtered, re-referenced to the average of channel TP9 and TP10. The MR gradient artifacts in the EEG data were corrected. The MR-denoised EEG data were then down-sampled to 500 Hz, and the cardioballistic signals from the ECG recording were used to adjust EEG signals via peak-detection algorithms in the BrainVision Analyzer software. Severe artifacts of EEG signal induced by muscle activities, environmental noise, eye movements, and blinking were manually removed to minimize their impacts on the subsequent analysis.

### Behavioral Data Analysis

We calculated SG and SS ratio of both scenarios to verify if each participant’s performance met the criterion. Behavioral characteristics of performance in the stop-signal task, including the go reaction time (Go-RT) and cSSD were analyzed with student’s *t* test (BFS vs. SBS). Furthermore, the stop-signal reaction time (SSRT) based on the horse-race model of stopping (Logan et al., 1984) was computed to represent one’s inhibitory ability. Since the stopping mechanism itself cannot be directly measured, the SSRT was calculated by subtracting SSD from the Go-RT. The inhibition function was computed as the number of SS trials divided by the number of all stop trials, and subjected to a two-way within-subject ANOVA to assess the effect of Scenarios (BFS vs. SBS), SSD (cSSD, cSSD ± 40 ms, and cSSD ± 80 ms), and their interaction.

### fMRI Data Analysis

The fMRI analysis was also completed in AFNI. Stimulus types and participant’s response conjointly determined four conditions for each scenario, including SG, SS, FS and fail go. The first-level statistical analysis for each participant was carried out in a general linear model (GLM) by convolving the onset of go stimulus in the SG, SS, and FS conditions, respectively, with a canonical hemodynamic response function (the BLOCK function in 3dDeconvolve of AFNI). Here the effects of interest are inhibitory control and error detection. The active brain areas for inhibitory control was defined by the contrast between SG and SS; on the other hand, the active brain regions for error detection was defined by the contrast between SS and FS. The scenario effect of inhibitory control and error detection were examined by comparing the “difference of difference”, namely (SS − SG)_BFS_ − (SS − SG)_SBS_ and (FS − SS)_BFS_ − (FS − SS)_SBS_, respectively. In the second level analysis, the between-scenario differences were analyzed with a linear mixed-effect model (3dMEMA), and the whole-brain type I error was controlled at a cluster threshold (alpha) of 0.05 via Monte Carlo simulation (3dClustSim).

To more sensitively detect activations associated with inhibitory control and error detection, we also carried out region of interest (ROI) analysis by both adopting ROIs related to stop-signal task in the literature (literature-based ROIs) and by selecting ROIs surviving the whole-brain analysis from the inhibitory control and error detection contrasts, respectively, regardless of scenarios (empirical-based ROIs). For the empirical-based ROIs, the leave-one-subject-out (LOSO) method (Esterman et al., 2010) was applied to extract the GLM coefficients, and the differences between scenarios were statistically assessed. It turned out the literature-based ROIs did not yield any significant difference between scenarios and will not be further described. On the other hand, six ROIs empirically identified from the whole brain analysis of inhibitory control and error detection, respectively, regardless of scenarios were analyzed to verify the between scenario difference. Empirical-based ROIs for inhibitory control included rIFG, left insula, preSMA, left inferior parietal gyrus (IPG), right middle occipital gyrus (rMOG) and left MOG. ROIs for error detection included right middle frontal gyrus (rMFG), left IFG, right IPG, right superior temporal gyrus (STG), right inferior occipital gyrus (IOG) and left MOG. The empirical MNI coordinates of inhibitory control and error detection were listed in the Supplementary Materials Table 1, while the literature-based ROIs were listed in the Supplementary Materials Table 2.

### EEG Data Analysis

**Figure 2:**
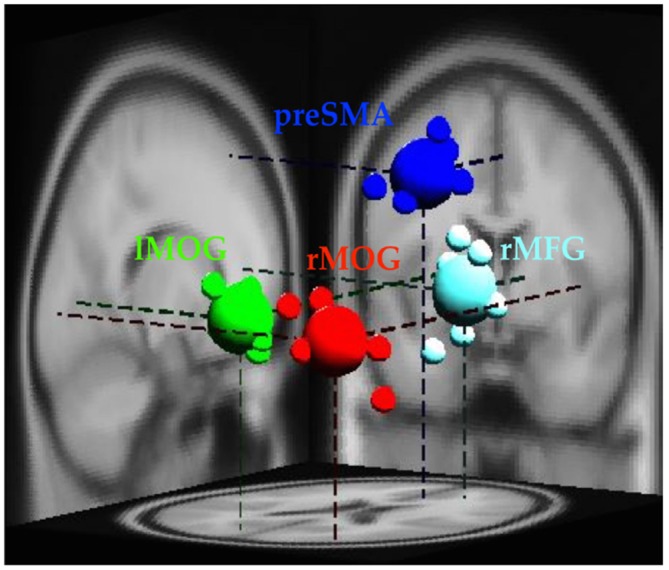
**Clusters of dipole locations for the analysis of EEG dynamics.** PreSMA and rMFG are for of inhibitory control and error detection, whereas lMOG and rMOG are used for processing visual stimul. Small spheres indicate individual participant’s dipole location, while large spheres indicate diploe locations of each cluster. lMOG, Left middle occipital gyrus; rMOG, Right middle occipital gyrus; preSMA, Pre-supplementary motor area; rMFG, Right middle frontal gyrus.

The EEG analysis was completed in EEGLab. Independent Component Analysis (ICA; Makeig et al., 1996; Delorme and Makeig, 2004) was used to separate out temporally independent time course of the activation of which dipole source location (Oostenveld and Oostendorp, 2002) was localized in the brain of each participant for group analysis (cross-subject analysis). We removed artifact components manually and then performed component clustering based on *k*-means (*k* = 5) criteria and dipole-fitting coordinates to identify the most representative clusters. The value of *k* was determined both by considering potential number of sources associated with the stop-signal task, and the number of ROIs identified in the fMRI results. One of the five resultant clusters was excluded because less than 70% of participants have it. Therefore, four clusters (preSMA, rMFG, and bilateral MOGs) and their dipole locations were identified (see Figure 2) to investigate brain dynamics following the go events and the subsequent stop events. Note that the preSMA and bilateral MOG clusters were in anatomical proximity of the inhibitory control ROIs of fMRI results, and the rMFG and the left MOG cluster was close to the error detection ROIs of fMRI results.

Each epoch was separately transformed into the time-frequency domain using the event-related spectral perturbation (ERSP) routine (Delorme and Makeig, 2004). Three conditions, namely SG, SS and FS, were identified as the effect of interest. The baseline was defined as the signals between −0.5 and 0 s before Go-stimulus for comparing response magnitudes of corresponding epochs. A two-way Scenario × Condition ANOVA was conducted on the baseline data to verify whether they are equivalent across scenarios and conditions. We have explored not only the power spectrum of each condition, but also the power spectrum of inhibitory control and error detection, respectively, in each scenario which was also done in the fMRI analyses.

## Results

### Behavioral Results

**Figure 3:**
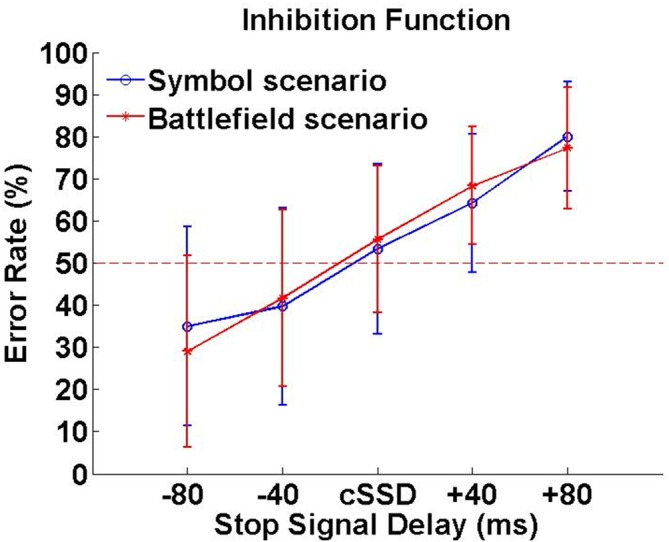
**Inhibition function.** Error rates (%) were calculated by dividing number unsuccessful stop trials with all stop trials under each SSD.

In SBS, the Go-RT, cSSD, SSRT, SG ratio and SS ratio of SBS were 425 ± 62 ms, 188 ± 50 ms, 240 ± 60 ms, 94.0 ± 7.6% and 45.6 ± 16.5%, respectively. In BFS, the Go-RT, cSSD, SSRT, SG ratio and SS ratio of SBS (BFS) were 422 ± 58 ms, 195 ± 68 ms, 230 ± 53 ms, 93.0 ± 10.3% and 45.6 ± 13.6%, respectively. When compared between scenarios, none of these behavioral outcomes reached significance (all *p*s > 0.05; Figure 3). In addition, the averaged inhibition function approached 50% at cSSD and error rate level increased with the length of SSD (Figure 3).

### Imaging Results

#### Inhibitory Control

##### Whole Brain Analysis

**Figure 4:**
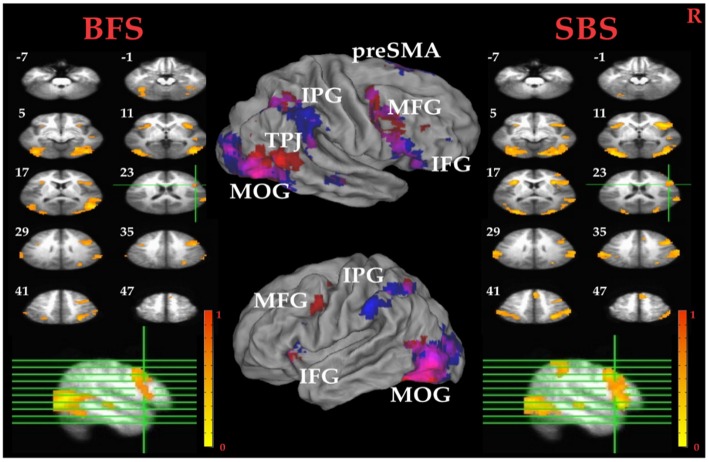
**Inhibitory control related brain areas.** All results were mapped onto a standard brain surface model in Caret (Van Essen et al., 2001). Left panel: horizontal sections under the BFS; middle panel: visualization of significant activations on the cortical surface for both scenarios (Red: BFS; Blue: symbol scenario [SBS]; Purple: overlap of both scenarios); right panel: horizontal slices under the SBS. The top-left number besides each slice indicate the *z*-axis. Right hemisphere is at the right side of the figure. Voxelwise statistical threshold was set at *p* < 0.0001, and cluster threshold alpha <0.01.

Tables 1A,B summarized brain regions that were more activated in the SS than in the SG condition, namely the inhibitory control component, under SBS and BFS, respectively. Figure 4 also shows these activations under the two scenarios conjointly so that overlapping brain regions are explicit. In SBS, the MOG and a few different frontal areas were activated in this contrast (see Table 1A and Figure 4 right panel). On the other hand, the brain areas activated by the BFS (see Table 1B and Figure 4 left panel) was similar to those in SBS (see the purple regions colored in purple in the Figure 4 middle panel). Moreover, when directly contrasting the two scenarios under inhibitory control, the only significant loci (BFS > SBS) fell within the right temporal-parietal junction (rTPJ; MNI: *x* = 48, *y* = −74, *z* = 11; cluster size = 39).

**Table 1:** **Brain regions more activated in (A) SS compared with SG under SBS, (B) SS compared with SG under BFS, (C) SS compared with FS under SBS, (D) SS compared with FS under BFS**.

Side	Region	BA	MNI coordinate (mm)			Cluster Size (Voxels)
			*X*	*Y*	*Z*
**(A) SS > SG under SBS**
R	Middle Frontal Gyrus	10	38	56	19	40
R	Superior Frontal Gyrus	8	2	26	59	159
R	Inferior Frontal Gyrus	47	48	16	1	844
L	Inferior Frontal Gyrus	13	−34	16	13	193
R	Superior Temporal Gyrus	21	50	−28	1	56
L	Inferior Parietal Gyrus	40	−60	−40	41	528
R	Middle Occipital Gyrus	19	44	−86	5	1752
L	Middle Occipital Gyrus	18	−34	−98	7	944
**(B) SS > SG under BFS**
R	Superior Frontal Gyrus	6	12	10	71	32
R	Middle Frontal Gyrus	6	50	−2	43	555
L	Inferior Frontal Gyrus	9	−40	−2	37	35
L	Inferior Frontal Gyrus	47	−30	20	1	95
R	Inferior Parietal Gyrus	40	60	−38	55	43
R	Superior Parietal Gyrus	7	32	−64	55	218
L	Superior Parietal Gyrus	7	−28	−68	55	31
R	Middle Occipital Gyrus	18	50	−74	11	1257
L	Inferior Occipital Gyrus	19	−42	−80	−5	694
**(C) SS > FS under SBS**
R	Inferior Frontal Gyrus	47	32	22	5	20
R	Inferior Frontal Gyrus	46	56	26	19	52
R	Middle Frontal Gyrus	8	48	2	49	21
L	Inferior Frontal Gyrus	45	−52	32	7	35
R	Postcentral Gyrus	4	66	−28	49	32
L	Middle Occipital Gyrus	18	−28	−82	−5	37
**(D) SS > FS under BFS**
R	Middle Frontal Gyrus	6	42	−2	49	21
L	Inferior Frontal Gyrus	45	−58	14	29	20
R	Inferior Parietal Gyrus	40	44	−40	59	89
L	Fusiform Gyrus		−34	−74	−7	216
R	Precuneus	7	26	−58	59	49
R	Middle Occipital Gyrus	19	32	−94	13	501

*Voxelwise threshold, p = 0.0001; cluster alpha < 0.01; BA, Brodmann Area; R, Right; L, Left*.

##### ROI Analysis

Pairwise *t* tests between the BFS and SBS in the six empirically defined ROIs for inhibitory control revealed significantly higher activation in BFS than in SBS at the left IPG (*t*_(32)_ = 2.4, *p* = 0.02) and rMOG (*t*_(32)_ = 2.5, *p* = 0.02).

#### Error Detection

##### Whole Brain Analysis

**Figure 5:**
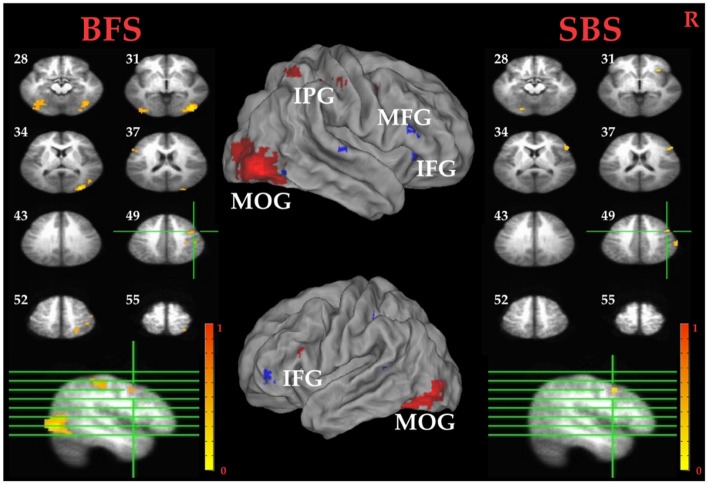
**Error detection related brain areas.** Left panel: horizontal sections under the BFS; Middle panel: visualization of significant activations on the cortical surface for both scenarios (Red: BFS; Blue: SBS; Purple: overlap of both scenarios); right panel: horizontal slices under the SBS. The top-left number besides each slice indicate the *z*-axis. Right hemisphere is at the right side of the figure. Voxelwise statistical threshold was set at *p* < 0.0001, and cluster threshold alpha <0.01.

Tables 1C,D summarized brain regions more activated in SS than fail stop under SBS and BFS, respectively. Figure 5 also shows these activations in both volumetric (left and right panels) and surface (middle panel) views as described in section “Inhibitory Control” for the inhibitory control. In SBS, the MOG, the bilateral IFG, the rMFG and right postcentral gyrus were activated in this contrast (see Table 1C and Figure 5 right panel). On the other hand, the rMFG, left IFG, the right IPG, the fusiform gyrus, the right precuneus and the rMOG were activated by the BFS (Table 1D and Figure 5 left panel). There was very few overlapping brain regions (purple regions in the middle panel of Figure 4). When directly contrasting the two scenarios under error detection, no region showed significant difference.

##### ROI Analysis

Paired *t* tests between the BFS and SBS in the six ROIs mentioned above revealed only significantly higher activation in BFS than in SBS at right IOG (*t*_(32)_ = 2.7, *p* = 0.01).

### EEG Results

**Figure 6:**
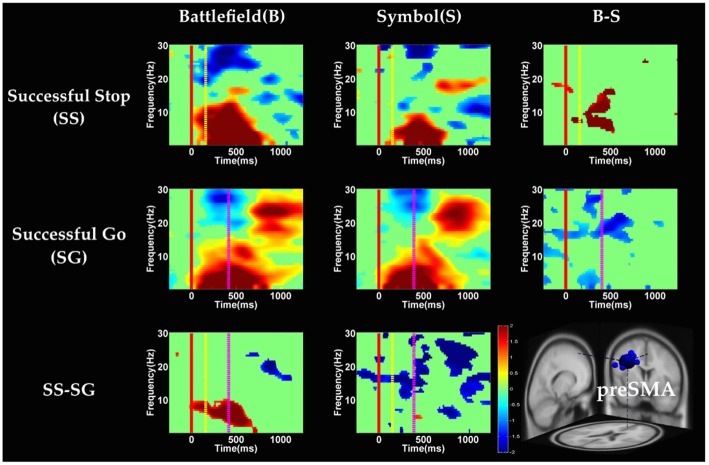
**The event-related spectral perturbation (ERSP) images of preSMA cluster under inhibitory control.** Red solid line: onset of the go stimulus; yellow dash line: onset of the stop signal; purple dash line: onset of response; color bars indicate the magnitude of the ERSPs; statistical threshold at *p* < 0.01.

**Figure 7:**
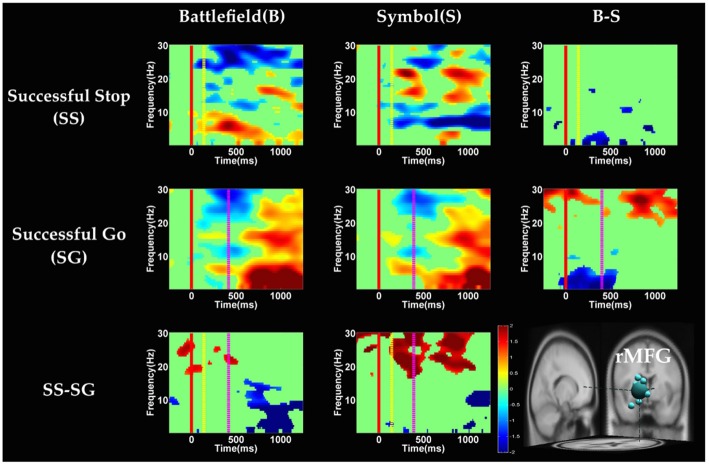
**The ERSP images of rMFG cluster under inhibitory control.** Red solid line: onset of the go stimulus; yellow dash line: onset of the stop signal; purple dash line: onset of response; color bars indicate the magnitude of the ERSPs; statistical threshold at *p* < 0.01.

**Figure 8:**
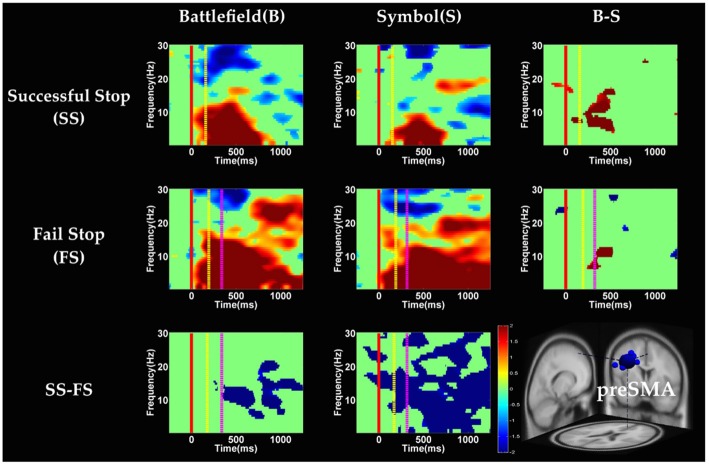
**The ERSP images of preSMA cluster under error detection.** Red solid line: onset of the go stimulus; yellow dash line: onset of the stop signal; purple dash line: onset of response; color bars indicate the magnitude of the ERSPs; statistical threshold at *p* < 0.01.

**Figure 9:**
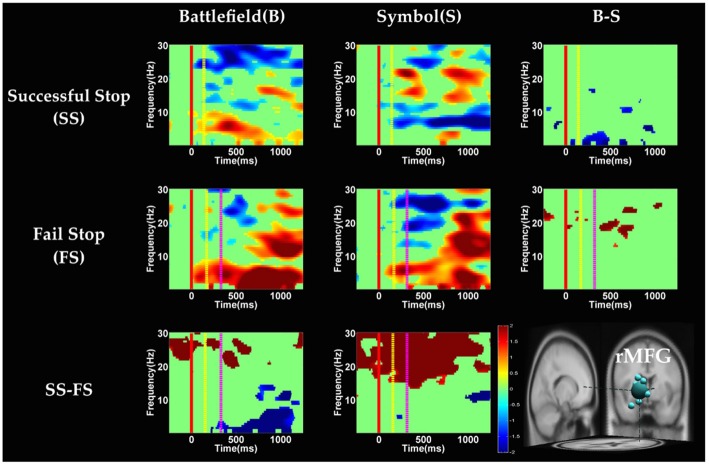
**The ERSP images of rMFG cluster under error detection.** Red solid line: onset of the go stimulus; yellow dash line: onset of the stop signal; purple dash line: onset of response; color bars indicate the magnitude of the ERSPs; statistical threshold at *p* < 0.01.

Figure 2 shows the four clusters (rMFG, preSMA, and bilateral MOGs) and their dipoles that fulfilled the cluster selection criteria (see “EEG Data Analysis” Section). Because the rMFG is considered as a crucial area for sustaining attention rather than stopping action and preSMA is considered as directly related to response inhibition, preSMA and rMFG were subject to the analysis at the time period when sustained attention and response inhibition were supposed to be ongoing. On the other hand, because bilateral MOGs were considered only relevant to visual perception that are relatively minor to the stop-signal task, their power ERSPs were analyzed at the time period of visual processing and described in the supplementary materials (Supplementary Figures 5, 6). For the rMFG and preSMA clusters described in the main text, the focus is on the contrasts for inhibitory control (i.e., SS vs. SG) and for error detection (i.e., FS vs. SS) in each scenario. The significant modulations within the individual conditions (i.e., SS, SG, and FS) can be found in Figures 6–9, which are mainly described in the following sections.

The baseline power of EEG oscillations were supposed to be equivalent between the SS and SG conditions as well as between the FS and SS conditions in both scenarios, because participants should be under similar state before the presentation of stimulus in each condition. Consistent with this assumption, one-way ANOVAs comparing SS and SG in BFS and SBS found no significant difference, and so did the one way ANOVAs comparing FS and SS. The analyses of baseline power are described in the Supplementary Figures 2, 4.

#### Inhibitory Control

Figures 6, 7 show the results of time-frequency analyses in preSMA and rMFG, respectively. In the preSMA component (Figure 6), the brain dynamics for inhibitory control can be examined by contrasting the SS and SG conditions. In this contrast, the burst of delta and theta band power was observed in BFS, whereas the suppression of alpha and beta band power was observed in SBS.

In the rMFG component (Figure 7), the brain dynamics for inhibitory control (SS vs. SG) showed delta, theta and alpha band power desynchronization after response in BFS, whereas beta band power was in synchronization after go stimulus in SBS.

#### Error Detection

In the preSMA component (Figure 8), the brain dynamics for error detection (SS vs. FS conditions) showed the suppression of theta and alpha band power in BFS; on the other hand, all frequency bands power of FS condition displayed much greater magnitude than SS in SBS.

In the rMFG component (Figure 9), the brain dynamics for error detection (SS vs. FS) showed that delta and theta band power were in desynchronization after response in the BFS, whereas beta band power was in synchronization after go stimulus in SBS.

## Discussion

The current study aims to compare inhibitory functions and the associated brain mechanisms underlying realistic and simplified scenarios. Based on the behavioral results, participants successfully performed the stop-signal task under BFS and SBS (the SG ratio was above 90% and SS ratio approached 50% for both scenarios). The SSRT has been suggested to be an indicator of one’s inhibitory ability (Band et al., 2003). Since SSRTs of the two scenarios do not differ, performance on response inhibition does not seem to be influenced by different scenarios one faces, likely due to highly adaptive nature of human’s inhibitory processing. The brain mechanisms for inhibition under the two scenarios can be compared on equivalent bases of behavioral performance.

To summarize, main findings in the fMRI and EEG data are as the following: in the whole-brain analysis of fMRI data, significant difference between the battlefield and SBSs was found only in rTPJ for inhibitory control, and no significant region was found for error detection. In the ROI analysis of fMRI data, significant difference between the two scenarios (BFS > SBS) was found in left IPG and rMOG for inhibitory control, and in right IOG for error detection. As for the EEG results, for inhibitory control in the preSMA, the burst of delta and theta band power was observed in BFS, whereas the suppression of alpha and beta band power was observed in SBS. In the rMFG, there were delta, theta and alpha band power desynchronization after response in BFS, and beta band power synchronization after go stimulus in SBS. For error detection in the preSMA, there was the suppression of theta and alpha band power in BFS, and broadband synchronization in SBS. In the rMFG, there were delta and theta band power desynchronization after response in the BFS, and there was beta band power synchronization after go stimulus in SBS.

### Neural Mechanisms of Inhibitory Control

The fMRI results show that, under the contrast of inhibitory control, the stop-signal task in BFS and SBS activate overlapped brain areas including preSMA, rIFG, bilateral IPG, bilateral MOG. All of these brain areas are either involved in target detection or attention to salient events (Corbetta and Shulman, 2002; Eckert et al., 2009; Menon and Uddin, 2010). Specifically, one of MOG functions is visual form perception and recognition (Grill-Spector and Malach, 2004), the parietal lobe is a crucial locus for spatial attention (Yantis et al., 2002), and the rIFG and preSMA show significant activation for contrast between SS and SG conditions (Boehler et al., 2010; Swick et al., 2011). While participants performed stop-signal task in both scenarios, we expect to observe stronger activations in BFS than SBS because BFS contains more complex visual information and may evoke other cognitive functions involved in the inhibitory network.

With respect to the main goal of the current study, when contrasting the inhibitory control component in both scenarios in the whole-brain analysis, we observe higher activation for the BFS in the rTPJ. The rTPJ has been implicated, together with the rIPG, in detecting behaviorally relevant salient events (Corbetta and Shulman, 2002; Husain and Nachev, 2007). Chang et al. (2013) uses transcranial magnetic stimulation (TMS) to interfere with bilateral TPJ to probe the function in attentional networks, and find that the rTPJ is critically involved in attentional reorienting. In addition, rTPJ is also involved in the “theory-of-mind” (ToM) network which includes the medial PFG, precuneus, right superior temporal sulcus and bilateral TPJ (Saxe and Kanwisher, 2003; Aichhorn et al., 2009). The ToM network increases metabolic activity when one thinks about other people’s thoughts. Koster-Hale et al. (2013) use multi-voxel pattern analysis to examine the difference between intentional and accidental harms on other people, and conclude that rTPJ is associated with moral judgments. In the current study, the rTPJ may serve one or a few of the functions mentioned above in BFS because the task involve shooting decision which may aim at innocent hostage.

There is a greater potential negative consequence of failing to stop a shooting response in the presence of an innocent hostage, which may actually decrease response impulsivity but yet still increase the level of activation of inhibitory systems. To verify this speculation with enhanced sensitivity, six brain areas are selected from the whole-brain analysis of inhibitory control that were localized by contrast orthogonal to the scenario effect, including the rIFG, preSMA, left insula, left IPG, rMOG and left MOG. The left IPG and rMOG show a greater activation in BFS. To relate the findings with the roles of these ROIs in previous studies, the left IPG has been implicated in tool manipulation (Ishibashi et al., 2011) or executive function (Kübler et al., 2003), which are both relevant in the current study because participants might have connected the task to firing with a gun (using a tool) to shoot terrorist in BFS. According to Slotnick et al. (2003), the reallocation of visual attention to external stimulus will result in an increase in occipital activation. In ROI results, the stronger activation of rMOG suggests that participants might have focused on terrorist and hostage and ignore the battlefield background. However, as BFS and SBS not only differed in their contextual information but also their visual complexity and emotional implications, the above conjectures need to be considered with caution.

With respect to the temporal dynamics, we first examine common findings in SS and SG conditions of both scenarios. In the preSMA source, there is a burst of each frequency band power except the beta band following the go stimulus, which lasted for 400–600 ms. This phenomenon is consistent with what was found after no-go and stop signal trials in previous studies (Schmiedt-Fehr and Basar-Eroglu, 2011; Huster et al., 2013). Because the preSMA is essential for the conversion from volitional thoughts to actions (Penfield and Welch, 1951; Fried et al., 1991), the beta band power has been generally considered as a marker of explicit responses. The event related desynchronization (ERD) of beta band occurs before and during response and then the event related synchronization (ERS) would follow actual response (Schulz et al., 2014). In the current experiment, the ERD of beta band occurs in SS and SG conditions of two scenarios likely because participants have already prepared to respond when they see the go stimuli; however, the ERS of beta band only occurs in SG condition of both scenarios because participants do not make actual response in the SS condition. Furthermore, the spectral perturbation of SS between BFS and SBS show that the power of theta-alpha band is much greater in BFS. According to Huster et al. (2013), the burst of frontal theta band power is associated with successful inhibition. In the current study, we observed synchronization of theta-alpha band power of SS under BFS and SBS in the preSMA. Furthermore, the theta-alpha band power in preSMA of BFS is higher than SBS, which suggests that the impulsivity in BFS is stronger than in SBS.

One thing worth noticing is that, in the preSMA brain source (Figure 6), there is no difference in the baseline power between scenarios, likely because each go stimulus may or may not be followed by a stop signal. This indicates that these two scenarios had the same baseline states when preparing for inhibiting prepotent response in the current trial regardless of stop signal. However, unlike in fMRI analysis, the rTPJ does not show significant differences between scenarios in the EEG analysis.

On the other hand, Swann et al. (2012) demonstrate that the power of 4–15 Hz is suppressed and beta band power would increase in right frontal lobe after the stop signal. Beta band power from the right frontal lobe may serve to compute coherence with preSMA. Therefore, they suggest that right frontal lobe monitors and detects the stop signal and then transfers the information to preSMA (coherent beta activity). This finding about the role of the right frontal lobe is similar to our results of SS of SBS at the rMFG, but not in the BFS. Perhaps the rMFG is involved in transferring information but not directly in inhibitory control so that different scenarios evoked different spectral perturbation. Finally, the spectral perturbation of two scenarios under bilateral MOG are similar, likely due to their similar roles in processing visual stimuli.

### Neural Mechanisms of Error Detection

In the whole-brain analysis, we observe higher activation in the IFG, MFG and MOG for the SS than the FS condition in both scenarios. These brain regions may reflect different cognitive functions in attention during visual processing, decision making, response execution and post-response processing (Iannaccone et al., 2015). Previous fMRI studies have indicated that attention neural network modulates visual cortical activation and facilitation of visual stimulus processing through inhibition of unattended stimulus information (Brefczynski and Deyoe, 1999; Smith et al., 2000; Slotnick et al., 2003). Although higher activation in MOG for error detection can be observed in both scenarios, we expect to observe stronger activations in BFS than SBS because BFS is a more complex situation requiring participants to correct their error and evoke other cognitive functions involved in inhibitory control (see also “Neural Mechanisms of Inhibitory Control” Section).

To verify the above speculation with improved sensitivity, rMFG, left IFG, right STG, right IPG, right IOG and left MOG were identified as ROIs from a contrast (SS—FS) orthogonal to the scenario effect in the whole-brain analysis. Only the rMOG shows greater activation in BFS than SBS. This result supports the idea that participants need to pay more attention to SS in BFS (Slotnick et al., 2003). Although we did not find significantly different activation of MFG between the two scenarios, the current findings still suggest that these middle and inferior frontal regions may differ in the post-response processes in the error detection (i.e., FS vs. SS). The middle and inferior frontal areas have been implicated in error detection and conflict monitoring (Braver et al., 2001; Menon et al., 2001; Rubia et al., 2003, 2005; Rushworth et al., 2004).

The current study also finds that both scenarios have stronger activation of MFG in error detection. The activation of MFG may reflect stronger performance monitoring after FS. Furthermore, we observed only the activation of fusiform gyrus in BFS (Table 1D) due to our stimuli design and may reflect face recognition (George et al., 1999).

With respect to the temporal dynamics, when analyzing the effect of scenarios, although ROI analysis in fMRI results reveal significant differences in the right IOG, we do not observe significant difference between scenarios in rMOG (Supplementary Figure 5). Although we do not find the effect of scenarios, we observe the suppression of theta band after response in error detection in both scenarios. Previous EEG studies have indicated that the oscillation of theta-alpha band is associated with the attention network (Fan et al., 2007). The current study finds that the suppression of theta band may be associated with the activation of right occipital gyrus for greater attention to the visual stimuli during SS when compared with FS. On the other hand, we explore the temporal dynamic in preSMA and rMFG brain source and observe the duration of burst of delta, theta and alpha band power in the FS condition were longer then SS condition in preSMA and rMFG. The prolonged duration of the FS condition may reflect error detection. The findings of previous EEG studies suggest that the burst of theta and alpha band power after response in the frontal lobe reflect error processing (Cavanagh and Frank, 2014; Cohen, 2015; Shou and Ding, 2015). Finally, this study reveals that the EEG oscillation of preSMA brain source is related to not only inhibitory control but also error detection.

## Conclusion

This study uses BFS to translate stop signal paradigm in simulated threatened situation and demonstrates that when human inhibits their action under threatened situation, the rTPJ is involved in the mediation of inhibitory control. The power of theta-alpha band under threatened situation is greater than normal situation that may be associated with the rising activation level of preSMA. Through over half a century of investigations on cognitive functions, significant amount of knowledge of basic cognitive processes has been acquired using stimuli with extremely simple configuration. From the behavioral performance of the current study we demonstrated that findings discovered with simple stimuli remains valid when carefully and comparably transformed into complex and realistic ones. At the meantime, additional brain regions relevant to the new configuration may be involved dynamically for the more complex stimuli, as can be identified from sources of signals differentially specialized in spatial and temporal resolutions. How these simultaneously recorded sources of signals (e.g., EEG and fMRI) are conjointly related to the valence, complexity, and motivational effects induced by scenes embedding the basic cognitive process remain an intriguing and important issue for future studies.

## Author Contributions

L-WK initiated the main idea of this study, designed the experiment and advised the EEG data analysis methods on the collecting fMRI-EEG data. Y-CS, RKC and Y-TC were performing the fMRI-EEG data collection and data analysis. ECC provided advises on modifying the experiment design and analyzing the fMRI data.

## Conflict of Interest Statement

The authors declare that the research was conducted in the absence of any commercial or financial relationships that could be construed as a potential conflict of interest.
