# Supplementary material for: Neural Mechanisms of Inhibitory Response in a Battlefield Scenario: A Simultaneous fMRI-EEG Study
Source: Front Hum Neurosci. 2016 May 2;10:185. doi: 10.3389/fnhum.2016.00185 (PMC4852198; doi:10.3389/fnhum.2016.00185)
Supplement: Supplementary file 1 [file Table_1.docx]

Supplementary Material

**Neural Mechanisms of Inhibitory Response in a Battlefield Scenario: a Simultaneous FMRI-EEG Study**

**Li-Wei Ko*, Yi-Cheng Shih, Rupesh Kumar Chikara, Ya-Ting Chuang, Erik C. Chang***

**Supplementary Table 1: ROIs coordinate for** **(A)** Inhibitory control, **(B)** Error detection

| **Side** | **Region** | **MNI coordinate (Standard Deviation)** | | | |
| --- | --- | --- | --- | --- | --- |
|  |  | **X** | | **Y** | **Z** |
| 1. Inhibitory control | | | | | |
| R | Inferior Frontal Gyrus | | 42.9(6.9) | 13.2(2.3) | 4.7(0.9) |
| R | preSMA | | 5.9(1.2) | 18.8(3.7) | 57.5(10.3) |
| L | Insula | | -31.6(6.8) | 15.3(2.9) | 7.7(7.7) |
| L | Inferior Parietal Gyrus | | -63.5(13.0) | -28.46(16.9) | 35.2(6.6) |
| R | Middle Occipital Gyrus | | 48.0(8.3) | -73.9(13.4) | 5.6(3.5) |
| L | Middle Occipital Gyrus | | -44.4(9.1) | -81.1(14.5) | 0.9(0.2) |
| 1. Error detection | | | | | |
| R | Middle Frontal Gyrus | | 44.9(8.1) | 1.9(0.4) | 44.7(8.0) |
| L | Inferior Frontal Gyrus | | -45.1(8.2) | 38.9(10.2) | 7.7(5.0) |
| R | Superior Temporal Gyrus | | 14.0(11.5) | -37.1(7.9) | 9.8(3.4) |
| R | Inferior Parietal Gyrus | | 41.4(7.8) | -37.0(8.6) | 52.8(9.8) |
| R | Inferior Occipital Gyrus | | 38.3(2.23) | -77.3(14.1) | -4.3(2.1) |
| L | Middle Occipital Gyrus | | -37.3(7.0) | -85.7(15.8) | 3.8(3.1) |

**Supplementary Table 2: Coordinates of literature-based ROIs**

| **Side** | **Region** | **MNI coordinate** | | | |
| --- | --- | --- | --- | --- | --- |
|  |  | **X** | | **Y** | **Z** |
| R | Inferior Frontal Gyrus | | 44 | 48 | -12 |
| L | preSMA | | -4 | 36 | 56 |
| L | Primary Motor Gyrus | | -36 | -8 | 52 |
| L | Caudate Head | | -10 | 8 | 5 |
| L | Subthalamic Nucleus | | -10 | -18 | 1 |
