# Supplementary material for: Neural Mechanisms of Inhibitory Response in a Battlefield Scenario: A Simultaneous fMRI-EEG Study
Source: Front Hum Neurosci. 2016 May 2;10:185. doi: 10.3389/fnhum.2016.00185 (PMC4852198; doi:10.3389/fnhum.2016.00185)
Supplement: Supplementary file 2 [file Presentation_1.pdf]

Supplementary Material - Figures

Neural Mechanisms of Inhibitory Response in a Battlefield Scenario: a Simultaneous FMRI-EEG Study

Li-Wei Ko*, Yi-Cheng Shih, Rupesh Kumar Chikara, Ya-Ting Chuang, Erik C. Chang*

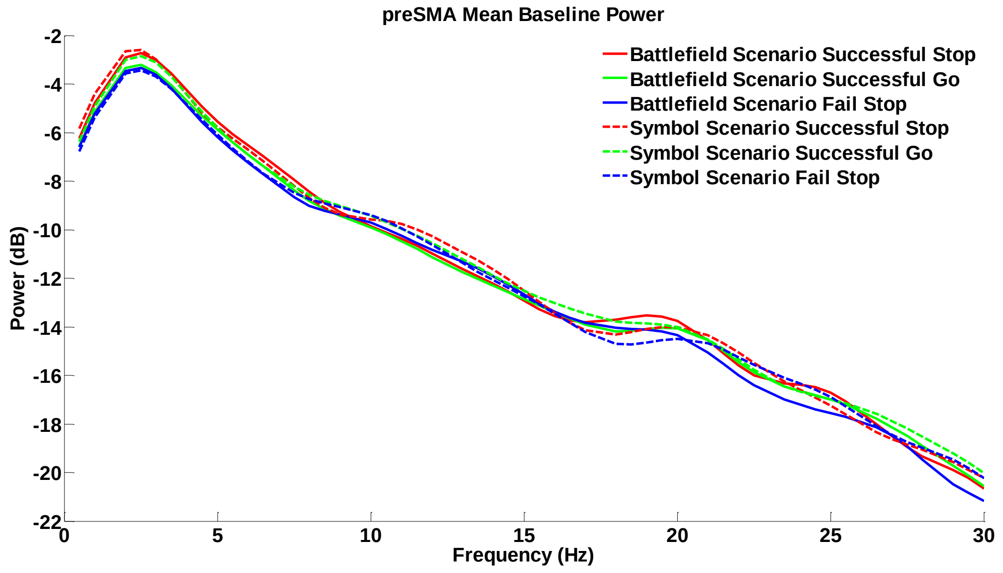

Supplementary Figure 1: Mean Baseline power of successful stop (SS), successful go (SG) and fail stop (FS) under preSMA.

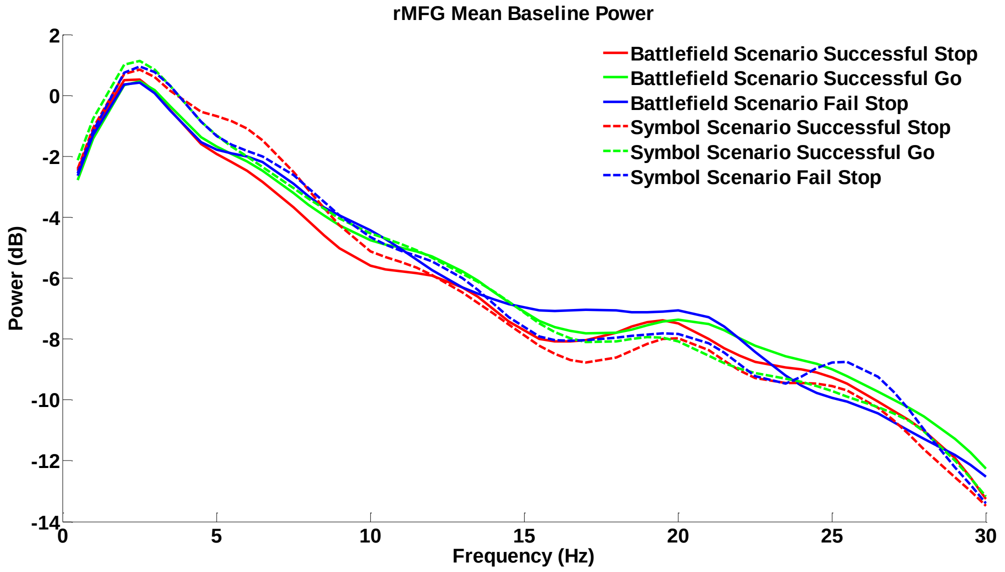

Supplementary Figure 2: Mean Baseline power of successful stop (SS), successful go (SG) and fail stop (FS) under right MFG.

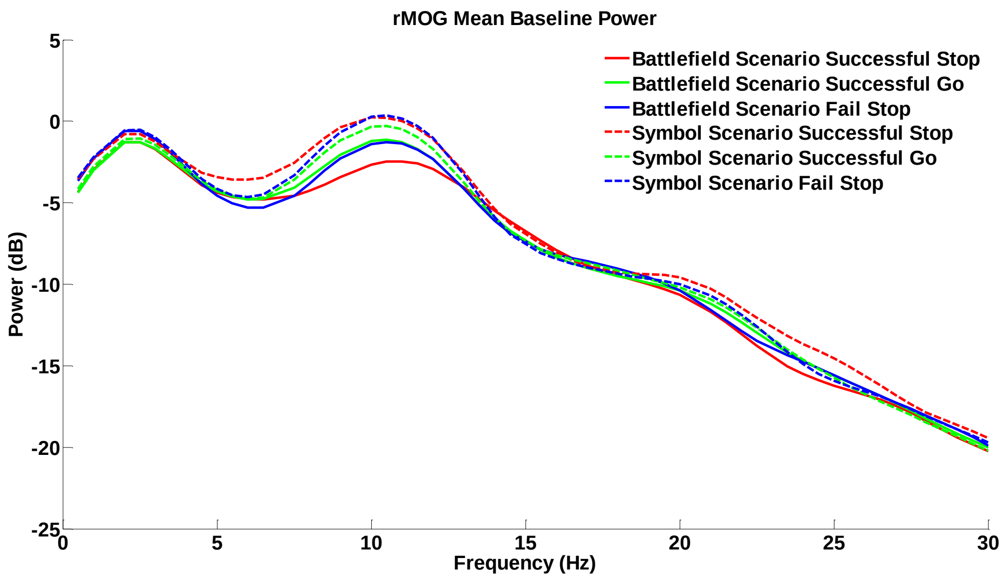

Supplementary Figure 3: Mean Baseline power of successful stop (SS), successful go (SG) and fail stop (FS) under right MOG.

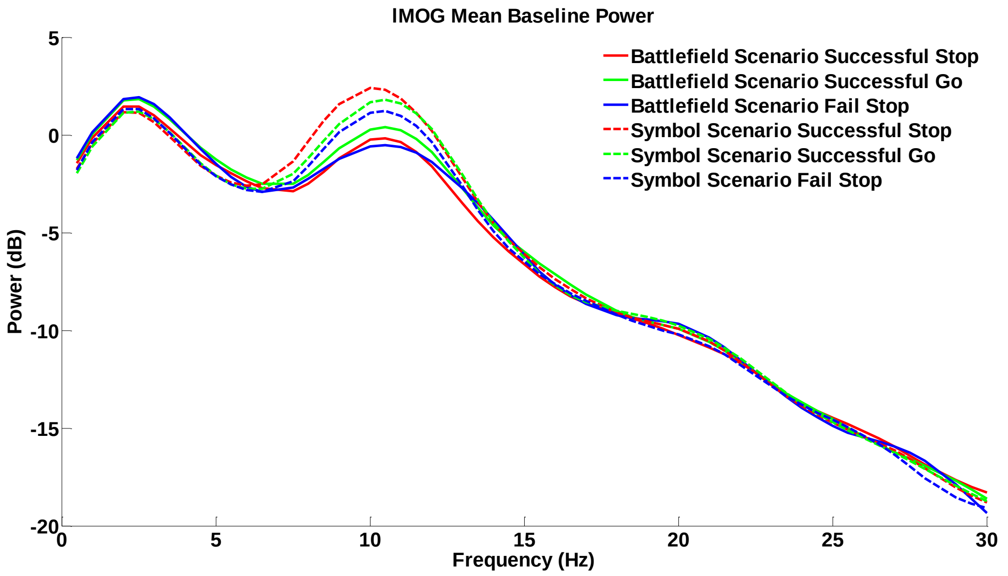

Supplementary Figure 4: Mean Baseline power of successful stop (SS), successful go (SG) and fail stop (FS) under left MOG.

EEG results of bilateral MOGs (middle occipital gyrus)

Inhibitory control. Supplementary Figure 5 and 6 showed the results of the inhibitory control contrast for bilateral MOGs. The power of delta, theta and alpha band were significantly increasing after the go stimulus and then alpha and beta band power were significantly decreasing followed by the increasing power of delta, theta and alpha band in successful-stop and successful-go conditions in both scenarios. To examine the effect of scenarios, BFS and SBS were contrasted, and the results showed that alpha band power of BFS was much greater than SBS in successful-go; however, there was rarely any significant difference in the successful-stop condition.

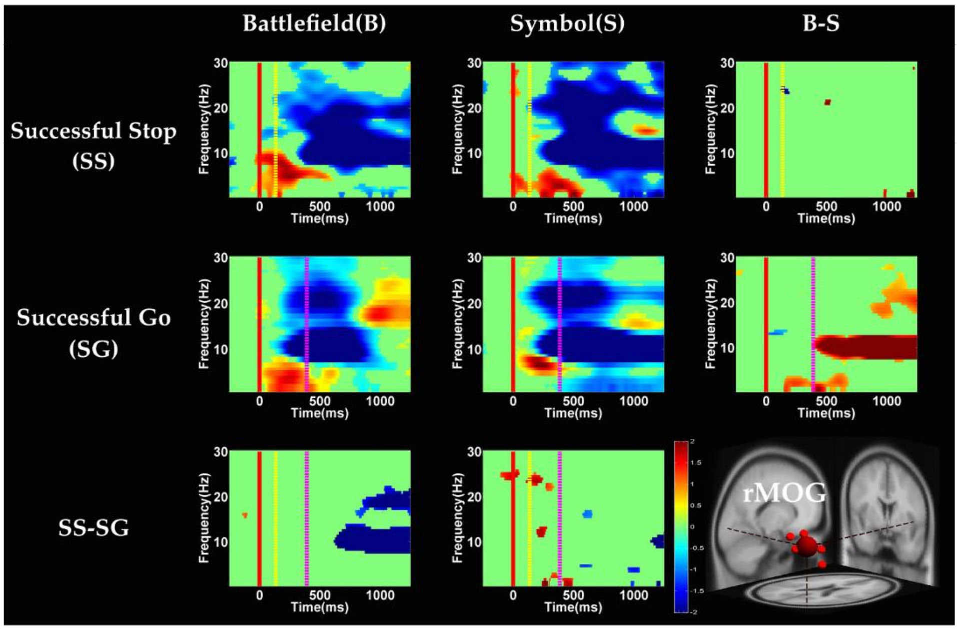

Supplementary Figure 5: The ERSP images of right MOG cluster for processing visual stimuli under inhibitory control. Red solid line: onset of the go stimulus; yellow dash line: onset of the stop signal; purple dash line: onset of response; color bars indicate the magnitude of the ERSPs; statistical threshold at $p < 0.01$.

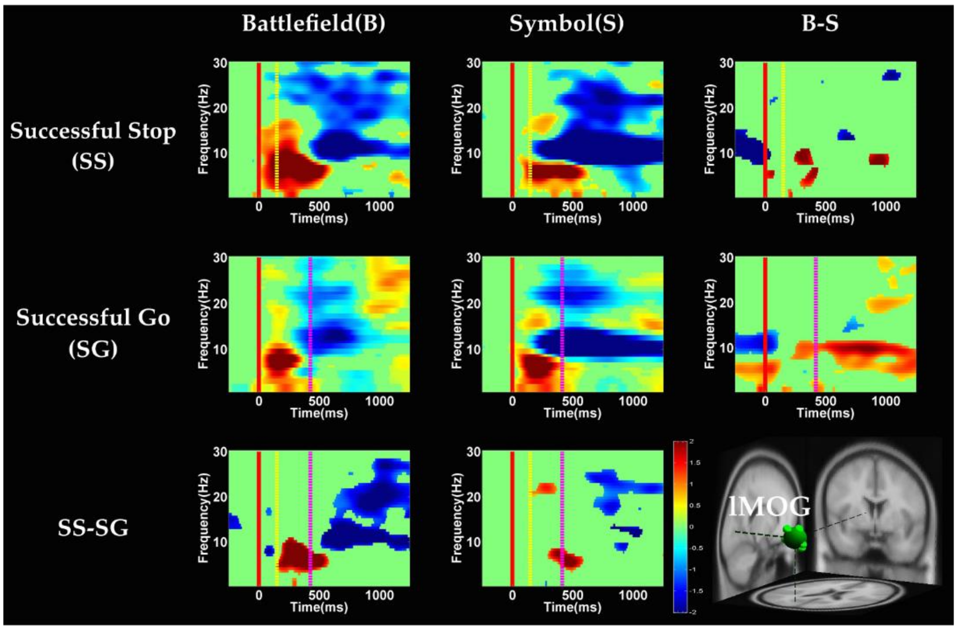

Supplementary Figure 6: The ERSP images of left MOG cluster for processing visual stimuli under inhibitory control. Red solid line: onset of the go stimulus; yellow dash line: onset of the stop signal; purple dash line: onset of response; color bars indicate the magnitude of the ERSPs; statistical threshold at $p < 0.01$.

Error detection. Supplementary Figure 7 and 8 illustrate the results of the error detection contrast for bilateral MOGs . The power of delta, theta and alpha band were significant increasing after the go stimulus and then alpha and beta band power were significantly decreasing followed by the increasing power of delta, theta and alpha band in fail-stop conditions in both scenarios. To examine the effect of scenario, BFS and SBS were contrasted, and the results showed that there was rarely any significant difference in the fail-stop condition.

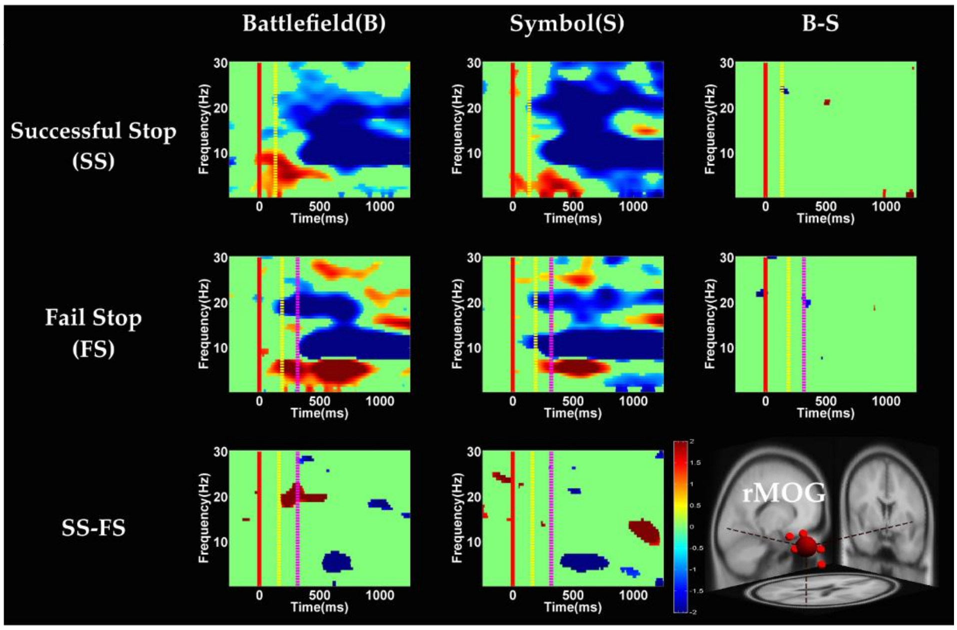

Supplementary Figure 7: The ERSP images of right MOG cluster for processing visual stimuli under error detection. Red solid line: onset of the go stimulus; yellow dash line: onset of the stop signal; purple dash line: onset of response; color bars indicate the magnitude of the ERSPs; statistical threshold at $p < 0.01$.

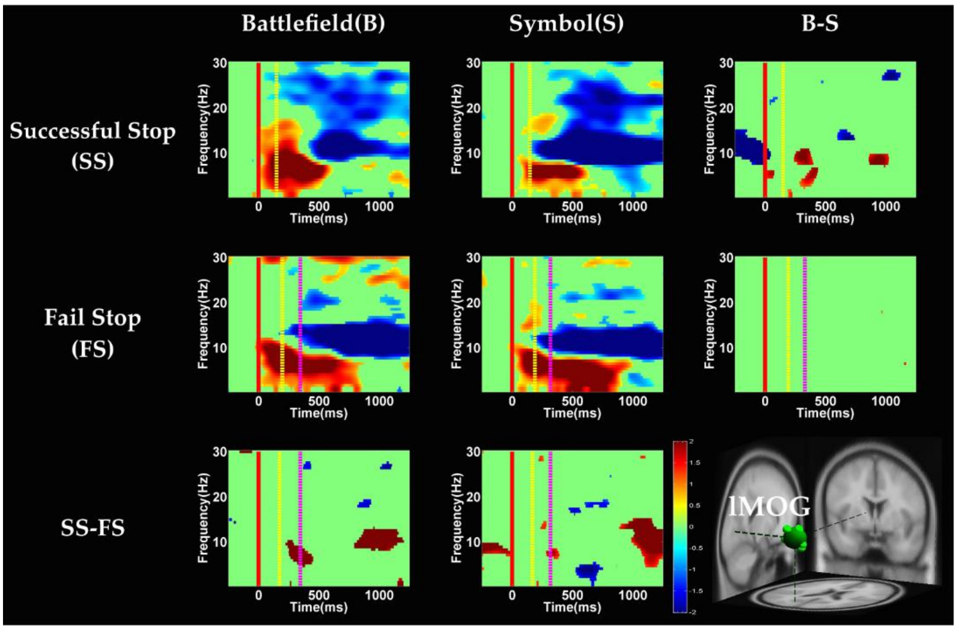

Supplementary Figure 8: The ERSP images of left MOG cluster for processing visual stimuli under error detection. Red solid line: onset of the go stimulus; yellow dash line: onset of the stop signal; purple dash line: onset of response; color bars indicate the magnitude of the ERSPs; statistical threshold at $p < 0.01$.
